# 

**Published:** 1905-12

**Authors:** 


					﻿^^tfesCRfPTION $1.00
A TT A NT A	monthly.
JOURNAL-RECORD
OF MEDICINE........
i t t? | > <.
Successor to Atlanta Hedical and Surgical Journal, Estanjisnecf
and Southern Hedical Record,	hlttAi’S’Qr^E
Vol. VII.	Atlanta, Ga., December, 19oo? ’	*	*j$o. 9.
				

